# Fabrication of Nanochannels

**DOI:** 10.3390/ma8095304

**Published:** 2015-09-17

**Authors:** Yuqi Zhang, Xiang-Yu Kong, Loujun Gao, Ye Tian, Liping Wen, Lei Jiang

**Affiliations:** 1 College of Chemistry and Chemical Engineering, Yan’an University, Yan’an 716000, Shaanxi, China; E-Mails: yqzhang@iccas.ac.cn (Y.Z.); glj@yau.edu.cn (L.G.); 2 Laboratory of Bio-inspired Smart Interfacial Science, Technical Institute of Physics and Chemistry, Chinese Academy of Sciences, Beijing 100190, China; E-Mails: kongxiangyu@mail.ipc.ac.cn (X.-Y.K.); jianglei@iccas.ac.cn (L.J.); 3 Beijing National Laboratory for Molecular Sciences, Key Laboratory of Organic Solids, Institute of Chemistry, Chinese Academy of Sciences, Beijing 100190, China; E-Mail: tianyely@iccas.ac.cn

**Keywords:** 1D nanochannels, biomimetic nanodevices, ionic rectification, biotic materials, nanopore

## Abstract

Nature has inspired the fabrication of intelligent devices to meet the needs of the advanced community and better understand the imitation of biology. As a biomimetic nanodevice, nanochannels/nanopores aroused increasing interest because of their potential applications in nanofluidic fields. In this review, we have summarized some recent results mainly focused on the design and fabrication of one-dimensional nanochannels, which can be made of many materials, including polymers, inorganics, biotic materials, and composite materials. These nanochannels have some properties similar to biological channels, such as selectivity, voltage-dependent current fluctuations, ionic rectification current and ionic gating, *etc.* Therefore, they show great potential for the fields of biosensing, filtration, and energy conversions. These advances can not only help people to understand the living processes in nature, but also inspire scientists to develop novel nanodevices with better performance for mankind.

## 1. Introduction

In today’s industrial production and human life, manipulating nanostructure has become increasingly important because it has great significance for many chemical, electronic, and biological advances [1,2,3,4,5]. In the field of chemistry, the reaction rate of nanostructured catalysts increases by several times compared to that of conventional catalysts [6,7,8], due to the improved number of surface active centers of the nanostructures. In the field of electronics [9,10,11], new types of solar cells can be prepared with high photoelectric conversion efficiency using semiconductor nanostructures. In the field of biology, proteins [12,13,14], DNA [15,16,17], and RNA viruses [18,19] are all on the nanoscale. Clearly, nanostructures play important roles in both materials and life.

Nanochannel materials play a very important role. Compared to other structures, the channel shape has a large surface area, high porosity, low density, high permeability, and high adsorption properties, *etc.* It has been widely used for the separation and adsorption of hazardous gases, separation of materials, treatment of environmental pollution, and as a catalytic material and carrier; meanwhile, it is equally important in biological systems. In short, nanochannel materials and nanochannel-based devices have become a focus of current academic research [20,21,22,23,24,25,26,27,28,29].

Nowadays, in order to build smart devices for various applications, artificial nanochannel membranes fabricated from organic or inorganic materials have been well studied [30,31,32,33,34,35,36,37,38,39,40,41,42,43,44,45,46]. However, the fabricating of nanochannel materials is still complicated [47,48,49,50,51,52,53,54,55,56,57]. Based on the advantages of nanochannels and the difficulty of preparing them, we have investigated some general methods for preparing nanochannel membranes and we suggest four types of materials for the preparation of various artificial nanochannels: polymers, inorganics, biotic materials, and composite materials.

## 2. Fabrication of Nanochannels

### 2.1. Polymers

#### 2.1.1. Ion Track-Etching Method

**Figure 1:**
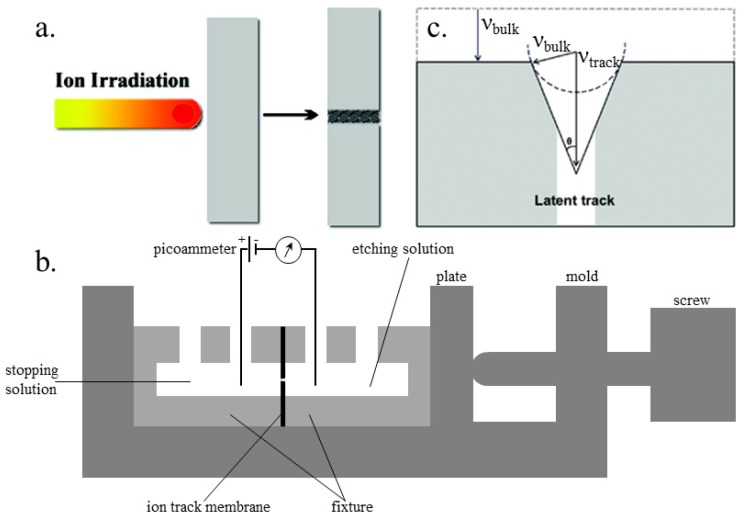
Schematic illustration of a nanochannel fabrication. (**a**) Origin of ion tracks. Reprinted with permission from reference [58]. Copyright 2008 American Chemical Society. (**b**) Cross-section of the electrolytic cell used for etching of ion track membranes. (**c**) An etched ion track. Reprinted with permission from reference [61]. Copyright 2012 the PCCP Owner Societies.

**Origin of latent tracks and fabrication of nanochannels:** The shape of the latent ion tracks can be maintained for a long time, formed by the energetic ions passing through insulating solids (Figure 1a) [58]. The passing ion transfers its energy to the bound electrons of the solid and releases a blast of secondary electrons, streaming radially along the ion path. With the distance from the ion path, the stored effect decreases rapidly [59]. Also, the track etch rate can be increased greatly by stockpiling the irradiated polymers in air.

The device for preparing the ion track polymer membranes is shown in Figure 1b [60]. During the etching process, the damaged area of a latent track is removed and converted into a concave channel. The etching rate, with which the latent track is dissolved, is marked as the track etch rate (ν_track_), while the etching rate of undamaged bulk material is referred to as the bulk etch rate (ν_bulk_). In the etching process, the track etch rate is obviously higher than the bulk etch rate. Therefore, the geometry of the fabricated nanochannel mainly relies on the ratio of track to bulk etch rate (ν_track_/ν_bulk_), which is further affected by the following four factors, (a) the etchant concentration; (b) the additives to the etchant; (c) temperature; and (d) the external applied voltage. Figure 1c schematically shows the correlation of ν_bulk_ and ν_track_. For better visibility, the scaling of the etch cone and the latent track is biased [61]. The etching of bulk material leads to a reduction of the foil thickness and a widening of the nanochannel perpendicular to the surface of the “etch-cone.” The removal of the track material with speed ν_track_ is responsible for the breakthrough of the membrane. The cone angle θ can be considered as a function of ν_bulk_ and ν_track_, where sin θ = ν_bulk_/ν_track_. When the track etch rate is high enough, ν_track_ >> ν_bulk_, the cone angle is almost zero and results in cylindrical-shaped nanochannels.

#### 2.1.2. Fabrication of Nanochannels in Different Polymer Membranes

**Polyethylene terephthalate (PET):** A 12 μm thick PET film (Hostaphan RN12, Hoechst) is used where the film samples (circular in shape, diameter 30 mm) were irradiated with individual single-ions [60]. Before starting the etching process, the irradiated films were further subjected to ultra violet (UV) irradiation (exposure to each side for 60 min at the wavelength of 365 nm). A computer-controlled hermetically sealed electrolytic cell was used to etch the ion tracks and monitor their electrical properties. During the etching process, a selected constant voltage was applied to the cell while monitoring the electrical current through a picoammeter.

The etching procedure was carried out at room temperature by filling one compartment of the cell with 9 M NaOH. The other compartment of the cell was filled with 1 M KCl or with a mixture of 2 M KCl and 2 M HCOOH (1:1 by volume) that served as a neutralizing agent [61]. This leads to a narrow cone of several degrees opening angle. After reaching a preset value of the electric current, the etching was interrupted by adding the stopping medium. Inert Pt-electrodes were used during etching.

**Polyimide (PI):** We used 12.5 μm thick polyimide (PI) films (Kapton 50 HN, DuPont). Unlike the PET etching process, track etching of the PI foil was performed in sodium hypochlorite (NaClO) at 50 °C [62]. Previous studies on PI film demonstrated that a strongly basic pH value of the etchant and a high content of active chlorine can guarantee the formation of conical pores with large opening angles. Thus a NaClO solution of initially high pH value (12.6) with an active chlorine content of 13% was selected to conduct the etching process. The irradiated PI film is settled between two compartments of a conductivity cell and etched from one side. The other half of the cell was filled with 1 M potassium iodide (KI) solution as a stopping medium for the ClO^−^ ions of the etchant [61]. As soon as the etchant completely penetrated the film, the iodide ions reduced ClO^−^ to Cl^−^ ions:

ClO^−^ + 2H^+^ + 2I^−^ → I_2_ + Cl^−^ + H_2_O

Through this reaction, the etching process stopped instantly after the breakthrough, allowing the preparation of highly narrow pores. During etching, a voltage of 1 V was applied across the cell to monitor the electric current with inert Pt electrodes. This allowed fast determination of the breakthrough moment, indicating that the etching of the membrane was completed. To obtain nanopores, the etching was interrupted shortly after the breakthrough moment by washing out the etchant with water and KI. Longer etching led to a gradual increase of the pore opening, monitored by an increase in the current [63].

**Polycarbonate (PC):** To obtain a PC membrane with cylindrical pores, an irradiated sample with tracks was etched by 6 M sodium hydroxide solution (NaOH) at 60 °C for 16 min. The samples were treated in parallel with 6 M NaOH solution containing surfactant. The surfactant was sodium dodecylbenzene sulfonate (SDBS) (Chameleon, Osaka, Japan) with a concentration of 0.01 wt %. To study the rate of etching PC membrane, an experiment has been done. A part of the PC film sample was etched chemically without irradiation by accelerated ions (6 M NaOH, 60 °C, 60 or 120 min). This procedure induced the removal of the surface layer (about 1 or 2 μm thick from both sides), depending on the treatment time [61,64].

**Figure 2:**
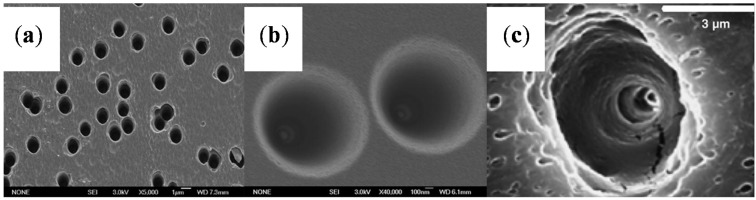
Scanning electron microscopy (SEM) images of the wide opening side on the polymer films. (**a**) The base side of nanochannels in polyethylene terephthalate (PET) membrane; the diameter was ~350 nm. Reprinted with permission from reference [61]. Copyright 2012 PCCP Owner Societies. (**b**) The base side of a nanochannel in track-etch polyimide (PI) membrane. Reprinted with permission from reference [65]. Copyright 2013 Royal Society of Chemistry. (**c**) Scanning electron micrographs of the base opening of track etched polycarbonate (PC) film. Reprinted with permission from reference [66]. Copyright 2006 John Wiley and Sons.

The results obtained from the polymer films (Table 1) are illustrated by the scanning electron microscopy (SEM) images in Figure 2 [61,65,66].

**Table 1:** Conditions of etching polymer membrane.

Polymer	Etchant	Stopping Solution	Temperature	Reference
PET (Conical shape)	9 M NaOH	1 M KCl or mixture of 2 M KCl and 2 M HCOOH (1:1 by volume)	~23 °C	[60,61]
PI (Conical shape)	NaClO solution of initially high pH value (12.6) with an active chlorine content of 13%	1 M KI	50 °C	[61,62,63]
PC (Cylindrical shape)	6 M NaOH (both sides)	-	60 °C	[61,64]

PET: Polyethylene terephthalate. PI: polyimide. PC: Polycarbonate.

**Figure 3:**
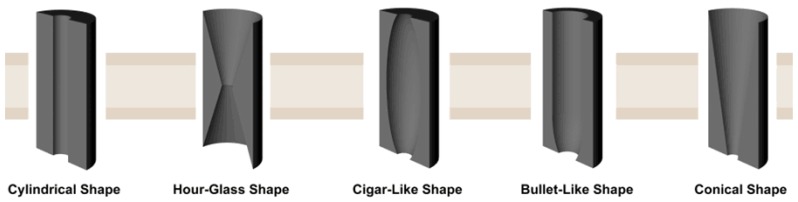
Artificial symmetric and asymmetric 1D nanochannels. Reprinted with permission from reference [67]. Copyright 2012 John Wiley and Sons.

**Shapes of the nanochannel in polymer membranes:** Diverse shaped nanochannels could be obtained by selectively etching the membrane under different conditions. There are five types of nanochannels: cylindrical, hourglass, cigar-like, bullet-like, and conical (Figure 3). Take the PET membranes, for instance; detailed etching conditions are listed below (Table 2).

**Table 2:** Conditions for obtaining nanochannels (PET) in shapes.

Shapes	Etchant	Stopping Solution	Temperature	Time	Ref.
Cylindrical	2 NaOH (Both sides)	-	50 °C	4 min	[68,69,70]
Hour-Glass	9 NaOH (Both sides)	-	~23 °C	-	[52,71,72,73]
Cigar-Like	6 M NaOH + 0.025% SDDD ^a^ (Both sides)	-	60 °C	16 min	[74,75,76]
Bullet-Like	6 M NaOH + 0.05% (*w*/*w*) Dowfax 2A1 ^b^	6 M NaOH	60 °C	-	[77,78,79]
Conical	9 M NaOH	1 M KCl + 1 M HCOOH	~23 °C	-	[60,80,81]

^a^ SDDD = Sodium Dodecyl Diphenyloxide Disulfonate; ^b^ Dowfax 2A1 = concentrated (approximately 45%) aqueous solution of sodium dodecyl diphenyloxide disulfonate (Dow Chemicals).

#### 2.1.3. Block Copolymer Self-Assembly Method Based on Phase Separation Processes

The nanochannel membranes are prepared by controlling the phase separation of block polymer solutions into two phases, one with a high polymer concentration and the other with a low polymer concentration. After phase separation, the phase with high polymer concentration solidifies shortly and forms the membrane. The performance of this membrane strongly depends on the formed morphology during phase separation and subsequent solidification [82].

#### 2.1.4. Fabrication of Nanochannels Using Different Block Copolymers

**Figure 4:**
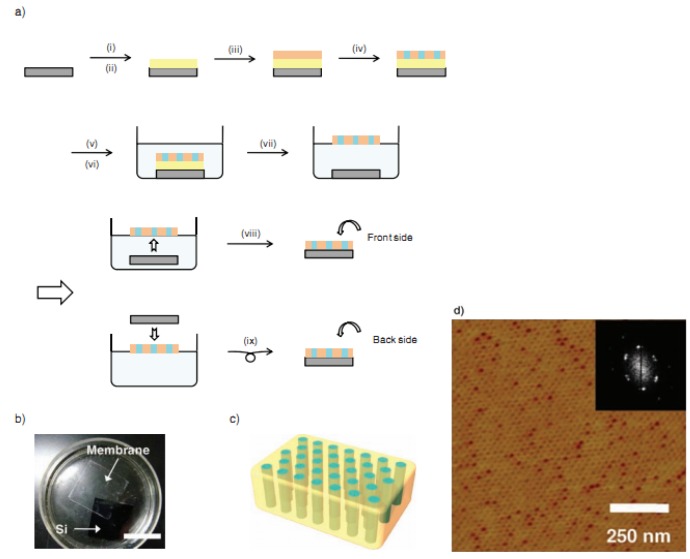
(**a**) Fabrication processes for the free-standing PEO_114_-b-PMA(Stb)_52_ membrane; (**b**) photograph of the free-standing membrane floating at the air-water interface. Si denotes a silicon wafer substrate. The scale bar represents 2 cm. (**c**) Illustration of the free-standing block copolymer membrane with the hexagonally arranged and perpendicularly aligned polyethylene oxide (PEO) transport channels. (**d**) Atomic force microscopy (AFM) phase image and its fast Fourier transform (FFT) pattern of the PEO_114_-b-PMA(Stb)_52_ film on a bare silicon wafer substrate. Reprinted with permission from reference [88]. Copyright 2011 John Wiley and Sons.

**Poly(ethylene oxide)-block-poly(methacrylate) bearing stilbene mesogens in the side chains (PEO_114_-b-PMA(Stb)_52_):** Fabrication of the block copolymer membrane based on the phase separation process [83,84,85,86,87] is shown in Figure 4 [88]. After spin-coating PEO_114_-b-PMA(Stb)_52_ (orange; 4 wt % CHCl_3_ solution, 2000 rpm, 30 s) onto a sacrificial cellulose acetate (CA) layer, we put the sample into an oven. Under a vacuum, it was annealed at 190 °C for 2 h, after which it finishes the phase separation process. According to the characterized results, even on the CA layer, PEO_114_-b-PMA(Stb)_52_ shows a highly ordered microphase-separated structure with hexagonally arranged cylinders. Remarkably, it is not necessary to regulate the surface energy to induce the microphase separation, such as precoating a random copolymer with an identical composition [89].

However, the prepared free-standing membrane is so brittle that, when dissolving the CA layer in acetone, it might be broken into many tiny fragments. Therefore, a new improvement is required to prepare more flexible free-standing membranes. As stilbene is a photofunctional moiety that undergoes two photochemical processes as follows: trans-to-cis photoisomerization followed by oxidative cyclization to afford a phenanthrene structure; and photodimerization by [2 + 2] photocycloaddition to form a cyclobutane ring [90]. When limited in organized assemblies, the stilbene moieties go through the latter predominantly because of the preferable configuration of neighboring stilbene moieties for the reaction [91].

**poly(styrene-block-4-vinylpyridine) (PS-P4VP):** As a fundamental method for obtaining block copolymer films, dip-coating is an important industrial technique [92,93,94,95] that is based on the phase separation process [96,97,98,99,100]. A method of preparing poly(styrene-b-4-vinylpyridine) films with dip-coating has been reported [101].

**Figure 5:**
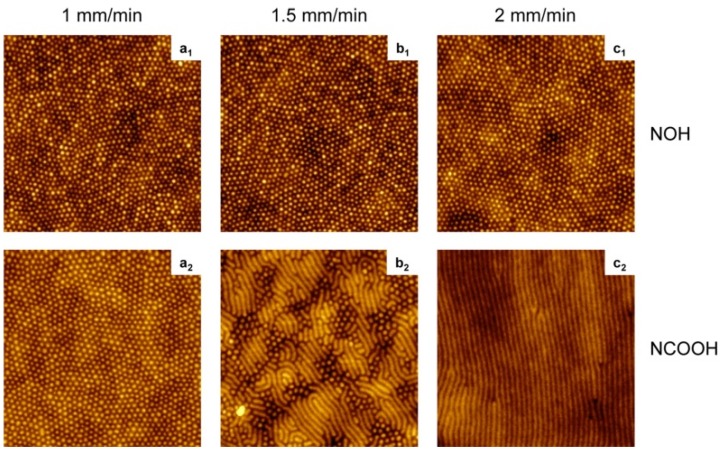
AFM images (2 × 2 μm^2^, *z* = 10 nm) of poly(styrene-block-4-vinylpyridine) (PS-P4VP) diblock copolymer films dip-coated at the rates indicated from tetrahydrofuran (THF) solutions containing SM = NOH or NCOOH at equimolar SM/VP ratios. Reprinted with permission from reference [101]. Copyright 2012 American Chemical Society.

To prepare the dip-coating solution, dissolve 25 mg of PS-P4VP in 5 mL of tetrahydrofuran (THF), so the concentration of the dip-coating solutions is 5 mg/ml. It is noteworthy that a required amount of small molecule (SM) for the desired SM/VP molar ratio needs to be added into the solution, too. The solution was stirred in closed vials on a heating plate at *ca.* 40 °C for several hours, followed by letting it stand until it cooled down to ambient temperature. Then it was filtered through 0.45 and 0.2 μm polytetrafluoroethylene (PTFE) filters. The whole dip-coating process should be implemented at a temperature of 21 °C, and no significant differences were found under different ambient humidity conditions. Silicon substrates were immersed vertically in the solution at a rate of 5 mm/min, paused for 30 s, and then seceded vertically from the solution at a controlled rate using a KSV 3000 Langmuir film balance. Finally, the films were dried in covered containers. The atomic force microscopy (AFM) topographic images of the PS–P4VP diblock copolymer films are shown in Figure 5.

### 2.2. Inorganics

#### 2.2.1. Particle Beam Sputtering Method

**Ion-beam sputtering method** **(Si_3_N_4_):** A way of fabricating devices at the micro/nano scale is reported by using low energy ion beams. Stein *et al.* [102] first reported a novel way for fabricating Si_3_N_4_ single nanopore by utilizing ion beam sculpting. This work plays a fundamental guiding role in the preparation of a solid molecular-scale hole or nanopore. Nanopores or nanochannels exist in living systems, where they serve as electric signal responsive components that regulate electric potential, ion flow, and molecular transport across cellular membranes. These nano-scale pores provide a great platform for localizing molecular-scale electrical junctions and switches. Furthermore, they could serve as a mask to prepare other tiny devices.

**Figure 6:**
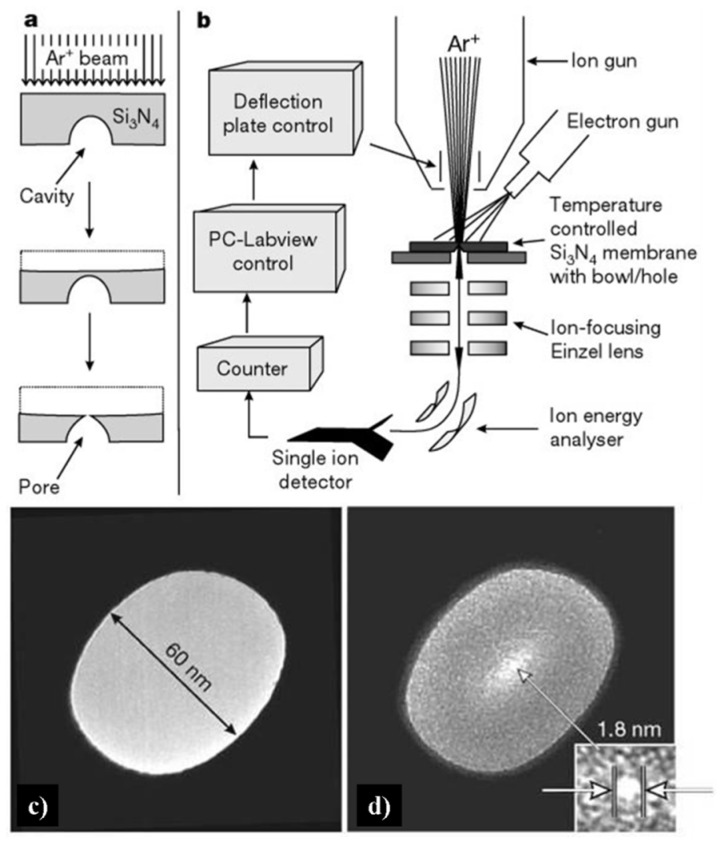
Strategy for making nanopores using argon ion-beam sputtering, and transmission electron microscopy (TEM) images of the nanopore in 500-nm Si_3_N_4_ membranes. (**a**) Sputtering removes material from a free-standing Si_3_N_4_ membrane with a cavity. (**b**) Feedback-controlled ion-beam sculpting apparatus housed in a high-vacuum chamber. (**c**) Initial 61 nm diameter pore made by focused ion beam (FIB). (**d**) The same sample after Ar^+^ ion-beam exposure. Reprinted with permission from reference [102]. Copyright 2001 Nature Publishing Group.

As shown in Figure 6, sputtering is a procedure in which massive ions with energies of several thousand electron-volts strike the sample surface and drive the sample atom to leave, resulting in atomic-scale erosion. A cavity containing flat Si_3_N_4_ surface endured Ar^+^ beam irradiating, forming a nanopore when the bottom of the bowl-shaped cavity was finally intercepted (Figure 6a, bottom). The details are as follows: A bowl-shaped cavity was produced at a Si_3_N_4_ membrane surface, which was held by a silicon frame (Figure 6b). Then a 3 keV Ar^+^ ion beam was applied at the back of the Si_3_N_4_ membrane until a nanometer-sized pore formed. Thus the transmembrane ionic current was proportional to the nanopore sizes, and the sculpting procedure can be monitored by applied current. The minimal size of as-prepared nanopore is evaluated as 2 nm. At room temperature, however, unexpected results occurred when the sculpture was applied. The pore remains closed even after lengthy ion beam irradiation. According to the transmission electron microscopy (TEM) images (Figure 6c,d), as the membrane thickness grew, the diameter of the hole reduced from 60 nm to 1.8 nm after the ion beam irradiation. Ziegler and Biersack [103] suggested that the ion beam energy could be deposited within 5 nm depth beneath the sample surface. Moreover, the modification of the Si_3_N_4_ nanopore would largely extend the application of the solid state nanochannel, such as regulating the transport of DNA by coating self-assembled monolayer (SAM) functional compounds [104].

**Figure 7:**
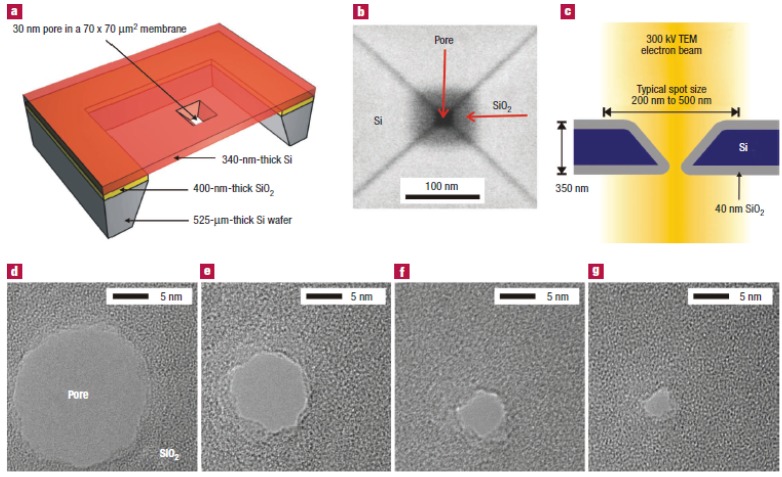
Fabrication of silicon oxide nanometer-sized pores. (**a**) Cross-section of the device. It consists of a 340 nm thick free-standing single-crystalline silicon membrane, supported by a 52 μm thick wafer etched by KOH. The membrane contains one or more submicrometer, pyramid-shaped pores, anisotropically etched with KOH from the top. (**b**) Top-view scanning electron micrograph of a nanofabricated pore after thermal oxidation. The pore is about 20 × 20 nm^2^ and is surrounded by an SiO_2_ layer of about 40 nm thickness. (**c**) Cross-section of the pore inside the electron microscope; (**d**–**g**) sequence of micrographs obtained during imaging of a silicon oxide pore in a TEM microscope. The electron irradiation causes the pore to shrink gradually to a size of about 3 nm. Reprinted with permission from reference [105]. Copyright 2003 Nature Publishing Group.

**Electron beam etching method (Si/SiO_2_):** In 2003, Storm and Dekker *et al.* [105] reported the fabrication of solid-state nanopores with single-nanometer precision using electron-beam lithography and reactive-ion etching. The fabrication of 20 to 200 nm pores in silicon oxide was built on Gribov’s earlier work [106]. Micromachining techniques were used to fabricate 70 × 70 μm^2^ free-standing silicon membranes in silicon-on-insulator (SOI) wafers with a top single-crystal silicon layer of 340 nm with crystal orientation <100>. The membranes were thermally oxidized on both sides with a SiO_2_ layer of 40 nm thickness. The squares with dimensions up to 500 nm in the SiO_2_ mask layer at the top were opened using electron-beam lithography and reactive-ion etching. Then, pyramid-shaped holes were etched using anisotropic KOH wet etching to strip the 40 nm oxide in buffered hydrogen fluoride and open up the pore in the silicon membrane (Figure 7a).The last processing step is a thermal oxidation to form a SiO_2_ surface layer with a thickness of 40 nm. Figure 7b shows a top-view scanning electron micrograph (from a Philips/FEI XL30S SEM) of the pore after the fabrication process. Each device used in the experiments reported here contains a silicon membrane with up to 400 pyramid-shaped holes with various dimensions from closed pores to pores of about 200 nm [107,108,109,110].

Furthermore, Dekker *et al.* [105] have reported a new technique to fine-tune the size of pores with nanometer precision. They only used a commercial transmission electron microscope (Philips CM-30UT), operated at an accelerating voltage of 300 kV in this experiment. Figure 7c shows a cross-section of a nanofabricated pore in the microscope. They found that an electron beam of intensity around 10^5^ to 10^7^ Am^−2^ caused pores to shrink if the initial diameter of the pore was about 50 nm or lower. Figure 7d–g shows the sequence of micrographs obtained during imaging of a silicon oxide pore in a TEM microscope. Remarkably, pores with initial dimensions of about 80 nm or higher had different dynamics. These pores expanded in size instead of the shrinking dynamics observed for small pores. The imaging mechanism of the microscope can be used to monitor the changes in pore diameter in real time.

**Figure 8:**
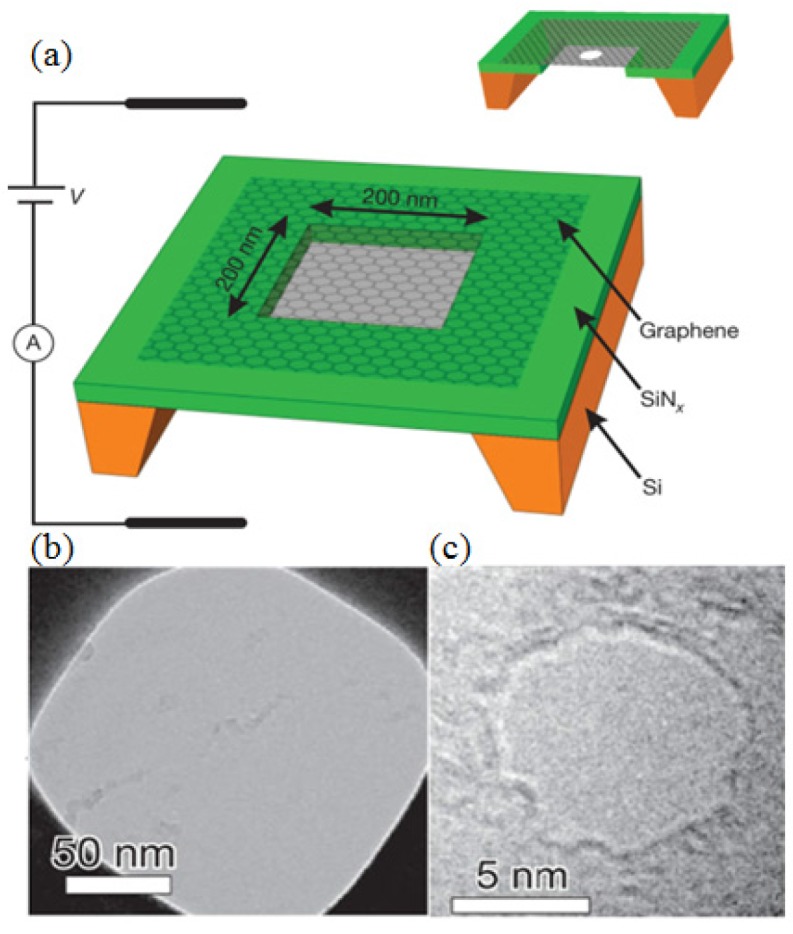
Diagram of the experiments and TEM images. (**a**) A graphene membrane was mounted over a 200 × 200 nm^2^ aperture in SiN_*x*_ suspended across a Si frame. Bottom: (**b**) a mounted graphene membrane; (**c**) the 8-nm pore. Reprinted with permission from reference [111]. Copyright 2010 Nature Publishing Group.

**Electron beam nanosculpting method (Graphene):** As shown in Figure 8a, the Si-SiN_*x*_ with synthesized graphene was initially coated with methyl-methacrylate/methacrylic acid (MMA-MAA) copolymer and then cut into 0.5 mm × 0.5 mm square pieces [111]. (The fabrication process of a graphene membrane can be found in [112,113].) To fabricate the single nanometer-sized pore through the graphene membrane, a focused electron beam in a JEOL 2010 FEG TEM operated at 200 kV accelerating voltage was used. After drilling the nanopore, the graphene nanopore chips were kept under a clean vacuum of ~10^−5^ torr for further usage [114,115,116,117,118]. The diameter of the pore, observed from TEM images, is 8 nm, as shown in Figure 8b,c. In addition, graphene-like two dimensional (2D) materials such as BN and MoS_2_ were employed as the substrates for the nanopore fabrication [119,120,121].

#### 2.2.2. Focused Ion Beam Direct Writing Method (Si_3_N_4_)

**Figure 9:**
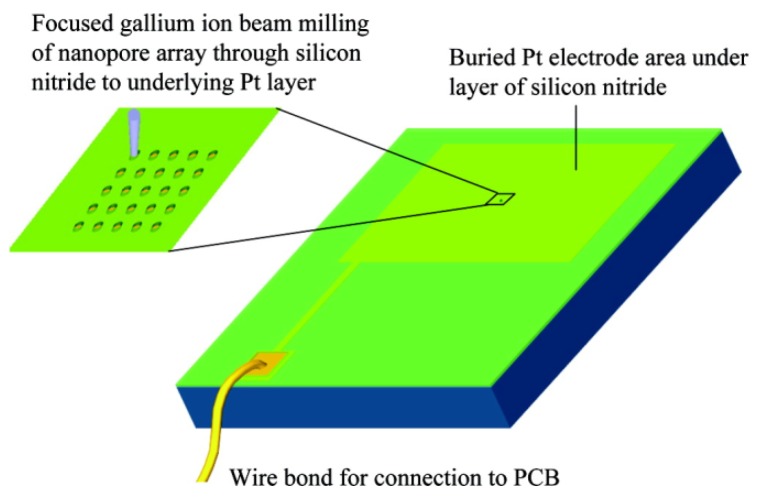
Schematic of the fabrication. Pt surface covered with silicon nitride is milled by a focused ion beam to open up nanopores through the underlying Pt. PCB: printed circuit board. Reprinted with permission from reference [123]. Copyright 2007 American Chemical Society.

**Figure 10:**
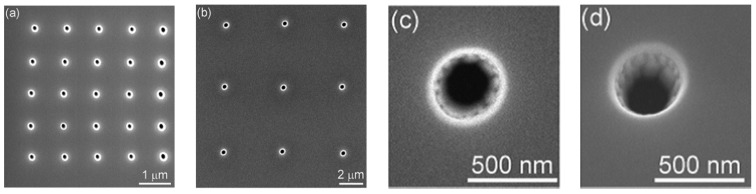
SEM images of nanopore electrode arrays and single nanopore electrodes: (**a**) 5 × 5 array; (**b**) 3 × 3 array; (**c**) single nanopore and (**d**) 10° tilted image of single nanopore shown in (**c**). Reprinted with permission from reference [123]. Copyright 2007 American Chemical Society.

Similar single nanometer channels, and even channel arrays, can be prepared by using focused ion beam direct writing [122]. However, the diameter of the hole is slightly bigger than that of the method described earlier, and this diameter is from 150 nm to 400 nm. Lanyon *et al.* created a Si_3_N_4_ insulating layer deposit on the platinum electrode surface. Then they used the focus of 30 keV Ga ion beam to etch and obtained a single channel or nanopore array (Figure 9) [123]. As shown, a focused ion beam (FIB) system (FEI Vectra 200DE, 30 keV Ga ions, 10 nm nominal spot diameter, 10 pA beam current) was employed for direct-writing nanoscale milling of the silicon nitride passivation layer. By using sequential milling, single nanopore and nanopore arrays with controlled pore diameters and pore-pore spacing were fabricated. The applications of this method had been reported in DNA analysis fields. [124,125]. Nanopore electrodes (array) and their structure characteristics were obtained by using field-emission SEM (6700 f SEM, Jeol Co., LTD.), which operated at a beam voltage between 3 and 10 kV (Figure 10) [123]. Also, the use of nanohole array could largely improve the response time in flow-through plasmonic sensing [126].

#### 2.2.3. Anodic Oxidation Method

**Figure 11:**
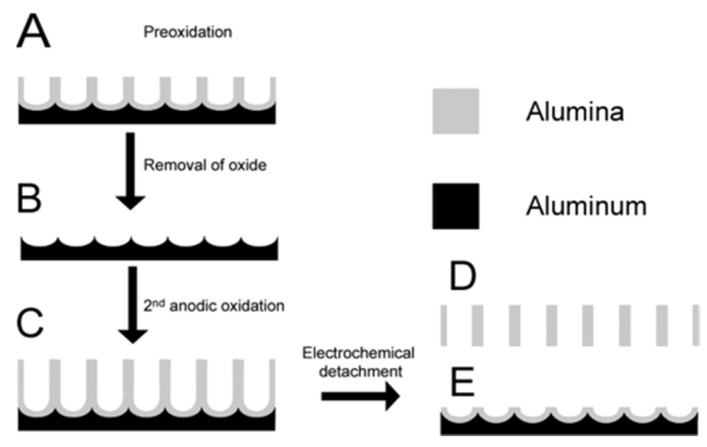
Schematic representation of the fabrication procedure for the formation of ordered and through-hole porous alumina membrane. (**A**) Formation of the porous alumina layer after the first anodic oxidation process; (**B**) removal of the porous alumina layer; (**C**) formation of the ordered porous alumina layer after the second anodic oxidation process; (**D**) free-standing porous anodic alumina (PAA); (**E**) the barrier layer structure on aluminum base after electrical detachment of the PAA. Reprinted with permission from reference [127]. Copyright 2004 American Chemical Society.

**Figure 12:**
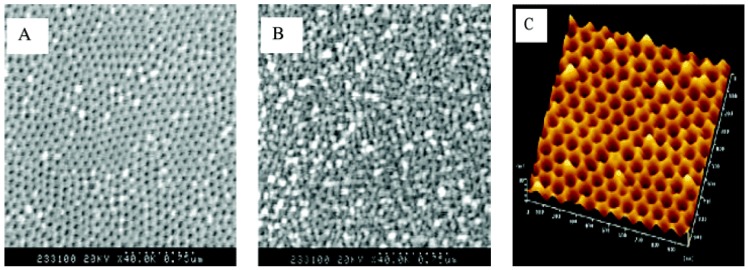
SEM images of the anodic alumina layer: (**A**) Top surface; (**B**) bottom surface. (**C**) AFM image of the top view of the barrier layer on the Al base. Reprinted with permission from reference [127]. Copyright 2004 American Chemical Society.

**Masuda’s two-step anodization process method (Al_2_O_3_):** The procedure to prepare a porous alumina membrane is schematically shown in Figure 11 [127]. In the first step, a clean aluminum sheet undergoes an anodic oxidization to form an alumina membrane (A) [128]. Then the preformed membrane is removed completely with a phosphoric acid solution to form a concave substrate with textured pattern (B) for the second anodic oxidation process. After another anodic oxidation, a well-ordered porous anodic alumina (PAA) membrane (C) with ordered pores is formed. This final film is then separated from the barrier layer by a voltage pulse of about 5 V for a short time to form a freestanding PAA membrane (D) and an alumina barrier layer covered with aluminum substrate (E). The SEM images of the anodic alumina layer are shown in Figure 12. The thickness of PAA membranes can be adjusted by the anodization time. For example, PAA membranes with nanochannel diameter of about 40 nm and 20 nm could be prepared by anodic oxidation of pure aluminum sheets in a 0.3 M oxalic acid electrolyte at a constant voltage of 50 V at 20 °C, and in a 0.2 M sulfuric acid at a constant voltage of 20 V at 10 °C, respectively [129].

**Figure 13:**
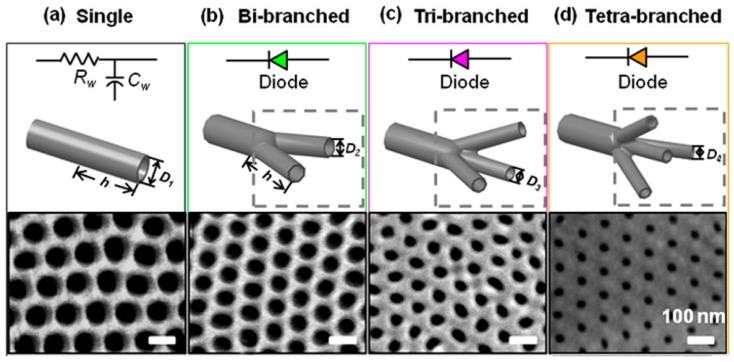
Equivalent circuit components, schematic diagrams, and SEM images of the final opening end of the prepared nanochannels; the scale bar is 100 nm. (**a**) alumina single nanochannel; (**b**) bi-branched alumina nanochannel; (**c**) tri-branched alumina nanochannel; and (**d**) tetra-branched alumina nanochannel. With an increasing number of branches, the pore diameters at the branch opening end decreased. Reprinted with permission from reference [132]. Copyright 2013 American Chemical Society.

**Branched alumina nanochannel:** Besides the penetrated cylinder PAA, the branched PAA can also be fabricated [130,131]. The number of branches in the final opening end could be controlled by tuning anode voltages [132,133]. Firstly, aluminum foils were anodized under a voltage of 50 V for 4 h. Then the resulting porous-oxide layer was removed with a 0.5 M phosphoric acid/0.2 M chromic acid mixture at 60 °C for 15 min. Next, different anodizing voltages could be used to control the number of branched nanochannels. The pore diameter is proportional to the anodizing voltage. Reducing the voltage by a factor of *n* (where *n* represents the number of the branches split from the primary stem) results in *n* times as many pores forming in the original total area of the oxide layer. In Figure 13, the anodized voltage was gradually reduced to 35, 28, and 25 V and the final channel segments formed bi-, tri-, or tetra-branched alumina nanochannels, respectively. The thickness of each nanochannel membrane was controlled by the anodization time independently. To fabricate the tetra-branched alumina nanochannel, a sulfuric acid electrolyte solution was used. Finally, the residual aluminum substrate was removed with saturated copper dichloride solution. The fabricated alumina nanochannels with different geometrical structures were characterized with a field-emission SEM (Figure 13, bottom).

**Figure 14:**
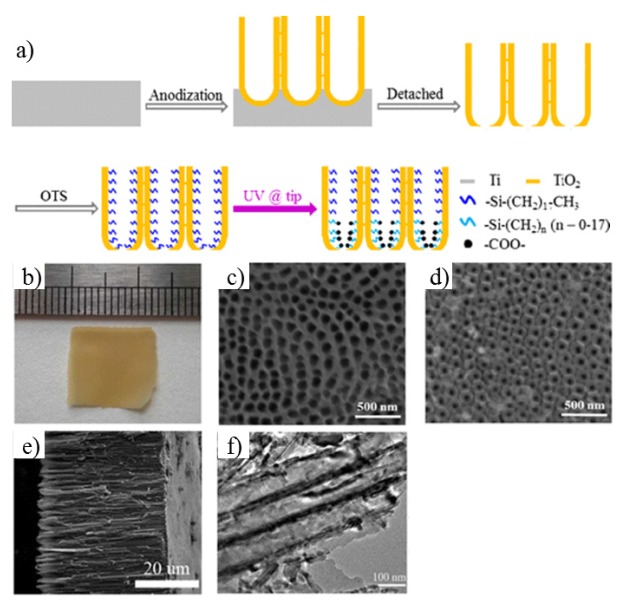
Fabrication of a TiO_2_ nanochannel membrane. (**a**) Schematic flow chart of the fabrication of artificial TiO_2_ nanotubular channels; (**b**) photograph of asymmetric TiO_2_ nanotubes fabricated under a voltage of 120 V. (**c**–**f**) The corresponding SEM images of (**c**) base side, (**d**) tip side, (**e**) cross section; (**f**) TEM image of TiO_2_ nanotubes. Reprinted with permission from reference [134]. Copyright 2013 American Chemical Society.

**Electrochemical anodization method to fabricate TiO_2_ nanochannel membrane:** The commonly used method is a three-step electrochemical anodization in ethylene glycol electrolyte-containing fluoride, from which people can prepare the self-standing asymmetric TiO_2_ nanotubes (Figure 14a) [134]. Here the Ti foil (Aldrich, purity = 99.7%) was used as a working electrode, while a Pt foil functioned as a counter electrode. All reactions were conducted in a water bath at room temperature. In the pretreatment for removing surface contaminations, the Ti foil was washed with ethanol, acetone, and distilled water in sequence by ultra-sonication. The clean Ti foil was anodized at 60 V for 1 h in an electrolyte consisting of 0.25 wt % ammonium fluoride, 2 vol % Milli-Q water, and ethylene glycol. The processed Ti foil was anodized at 60 V for 6 h to grow a TiO_2_ nanotubular layer. To prevent the TiO_2_ nanotubular layer from experiencing severe corrosion in the third-step (anodization), the layer was rinsed with isopropyl alcohol and dried. Then it was annealed at 200 °C for 3h in ambient conditions. Finally, the amorphous nanotubular layer was peeled off from the substrate with an electrolyte consisting of 0.5 wt % ammonium fluoride, 0.5 vol % Milli-Q water, and ethylene glycol at 120 V. In the meantime, it is to be crystallized to anatase phase under annealing at 450 °C for 3 h in ambient conditions with a heating rate of 1 °C·min^−1^ [135,136,137]. The images of the TiO_2_ membrane are shown in Figure 14b–f.

#### 2.2.4. Dielectric Breakdown Method

**Figure 15:**
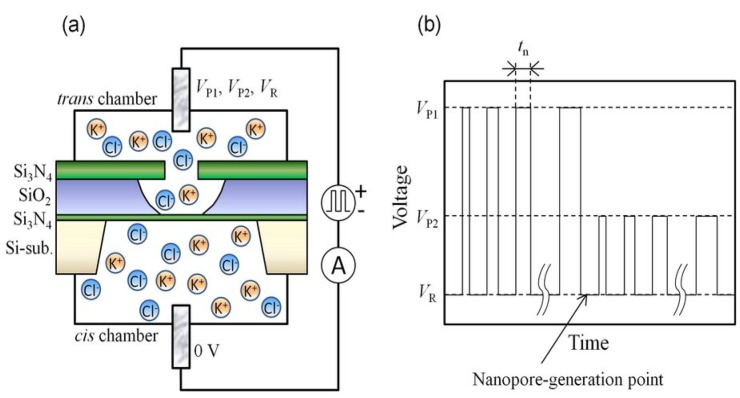
(**a**) Setup for multilevel pulse-voltage injection (MPVI). Cis and trans electrodes are immersed in both chambers and are connected to a voltage-pulse generator and an ammeter. (**b**) Pulse chart of MPVI, which uses three different voltages (*V*_P1_, *V*_P2_, and *V*_R_). *V*_P1_ is used to create a nanopore; *V*_P2_ is used to widen the nanopore to an intended size; and *V*_R_ is used to measure the current between the electrodes. Reprinted with permission from reference [138]. Copyright 2014 Nature Publishing Group.

Very recently, the dielectric breakdown method was developed for nanopore fabrication with sub-nanometer precision and controllable nanopore diameter. Yanagi and coworkers [138] demonstrated the sub-1 to 3 nm nanopore in 10 nm thick Si_3_N_4_ membranes by using the multilevel pulse-voltage injection (MPVI) technique. The dielectric breakdown is caused by the strong electric field produced by two conventional Ag/AgCl electrodes with pulsed voltage (Figure 15). Briggs *et al.* [139] demonstrated that a 2.0-nm and a 2.1-nm diameter nanopore fabricated by the dielectric breakdown method were capable of distinguishing single-stranded DNA *versus* double-stranded DNA, and that a 2.4-nm diameter nanopore could be used to investigate the overstretching transition in short dsDNA fragments. Moreover, the dielectric breakdown method was successfully applied in fabricating nanopores on graphene membranes [140], which showed its great potential in nanopore creation on different substrates.

#### 2.2.5. Electrochemical Etching Method (Glass)

**Figure 16:**
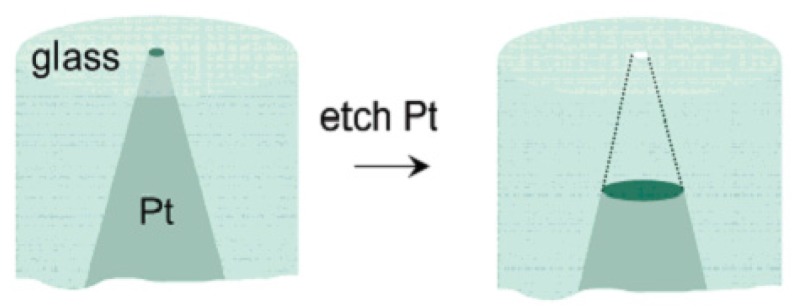
Fabrication of a nanopore electrode. Reprinted with permission from reference [141]. Copyright 2004 American Chemical Society.

**Figure 17:**
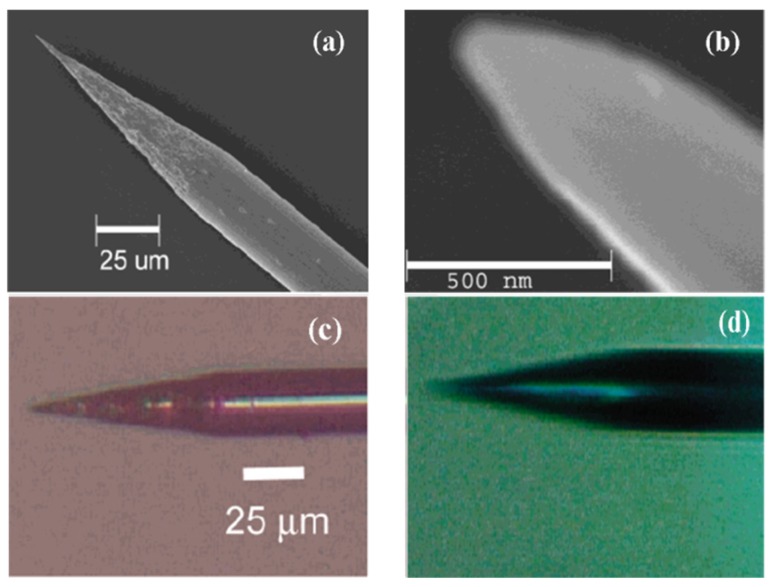
SEM images of a sharpened Pt wire (**a**,**b**). Optical microscopy images of an etched Pt tip before (**c**) and after (**d**) sealing it into glass. Reprinted with permission from reference [141]. Copyright 2004 American Chemical Society.

With the development of the solid-state electrochemical etching method, White and coworkers prepared a single nanopore electrode for the first time using platinum wire and a glass capillary [141]. The specific means were schematically presented in Figure 16: Firstly, sealing the pre-electrochemical etched platinum wire (Figure 17) to the glass capillary tube; and then polishing the bottom of the glass capillary until the diameter of the exposed platinum wire was in the range of 15–100 nm. After that, put the electrodes into the CaCl_2_ solution and etched platinum electrodes with an ac voltage of 5 V for a certain time. The diameter of the conical nanopore depends on the diameter of the unetched platinum [142,143].

#### 2.2.6. Mechanical Loading Method (Mica)

**Figure 18:**
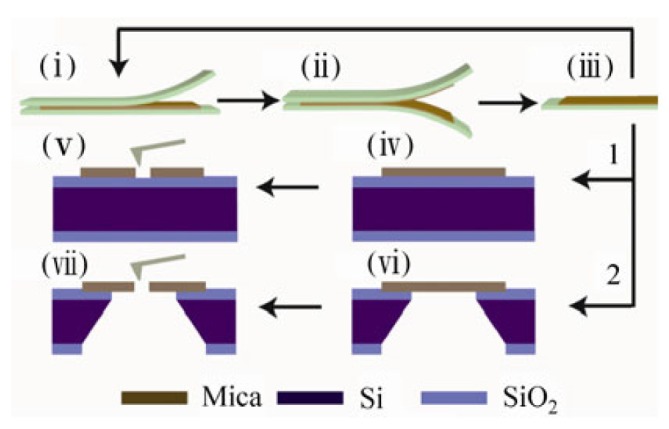
Schematic illustration of few-layer mica cleavage and conical nanopore fabrication. (**i**–**iii**) A freshly cleaved bulk muscovite mica sheet is attached to a sticky tape. Few-layer mica is prepared by repeatedly peeling off small flakes with other fresh pieces of tape.The peeled flakes can be transferred onto the top of either a solid silicon substrate (route 1) or a silicon window (route 2) for further characterization and processing. Nanopores can be fabricated in both solid (**iv**) and (**v**), and suspended (**vi**) and (**vii**) few-layer mica membranes by AFM processing with a certain mechanical load. Reprinted with permission from reference [144]. Copyright 2012 Springer.

**Figure 19:**
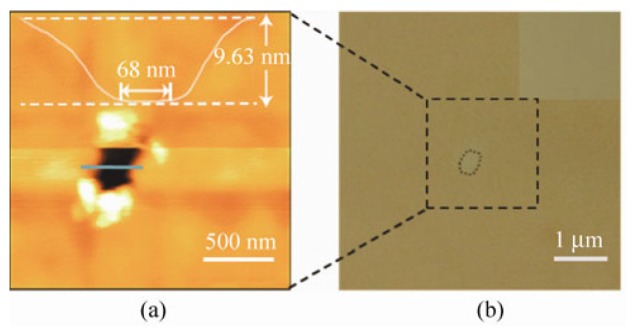
Images of the fabricated nanopore. (**a**) AFM image of an asymmetric ultra-thin nanopore processed with 250 nm × 250 nm scanning area on a 9.50 nm mica layer. The geometrically asymmetric height image shows the profile of the nanopore; (**b**) the same area viewed by optical microscopy. Reprinted with permission from reference [144]. Copyright 2012 Springer.

As shown in Figure 18, a mica membrane with a nanopore was fabricated with a freshly cleaved bulk muscovite mica sheet [144]. Few-layer mica was prepared by repeatedly peeling off small flakes with other fresh pieces of tape at least four times. Next, the peeled flakes were transferred onto the top of either a solid silicon substrate (route 1) or a silicon window (route 2) for further characterization and processing. After transfer onto the Si/SiO_2_ substrate, the few-layer mica flakes were characterized with optical microscopy, followed by AFM measurements to determine the actual thickness. Silicon wafers with a capping oxidized layer 0 nm, 250 nm, 300 nm or 500 nm thick were used as substrates for optical characterization. In optical characterization, the red green blue (RGB) values of five neighboring pixels were averaged. The mechanical load for AFM imaging was about 80 nN. Nanopores could be fabricated in both solid and suspended few-layer mica membranes by AFM processing with a certain mechanical load. The load on the AFM tip was 3863 nN. The obtained nanopores were geometrically asymmetric, like an inverted quadrangular frustum pyramid [145]. The optical and AFM images of the fabricated nanopore are shown in Figure 19.

In brief, diverse nanochannels were obtained in inorganic materials by utilizing particular nanofabrication technologies. The detailed methods and comments on the fabrication of nanochannels in inorganic membranes are listed in Table 3.

**Table 3:** Comments on various typical fabrication methods for nanochannels in inorganics.

Method	Materials	Comments	Ref.
Ion-beam sputtering method	Si_3_N_4_	The method could be useful for fabricating a variety of nanoscale semiconductor devices, as similar sculpting phenomena have been observed for geometries such as thin slits, trenches, and crosses, in several materials like SiO_2_, Si, and Al.	[74,75]
Electron beam etched method	Si/SiO_2_	Using the SOI-based process, it is straightforward to obtain this requirement with electron-beam lithography, and should be attainable even with optical lithography alone.	[43,46]
Electron beam nanosculpting method	Graphene	Nanometer-scale pores in the graphene were electron-beam-drilled in a 200-keV JEOL 2010 transmission electron microscope. The atomic thinness, stability, and electrical sensitivity of graphene motivate scientists to investigate the potential use of graphene membranes.	[51]
Focused ion beam direct writing method	Si_3_N_4_	FIB milling has great practical relevance for the fabrication of prototypes and their subsequent experimental evaluation prior to using more prolonged approaches to fabricate the engineered devices. The milling method creates a truncated cone-shaped pore, rather than a cylinder. Thus a model for diffusion-controlled current at a disk electrode at the base of such a truncated cone was developed.	[59]
Masuda’s two step anodization process method	Al_2_O_3_	A two-step oxidation process is enough for preparation of well-ordered pores. The present pore-opening process using short electrical oxidation for detaching the porous anodic alumina (PAA) film was used to improve the fabrication of anodic alumina with an array of nanopores.	[64]
Electrochemical anodization method	TiO_2_	Compared with previous artificial nanochannels, the new type of artificial nanochannel is more facile to fabricate and behaves as a diode that rectifies the ion transport, which also shows some other potential applications such as sensor and separation materials.	[67]
Electrochemical etched method	Glass	Electrodes with pore orifice radii less than 100 nm are relatively straightforward to fabricate using equipment and materials commonly found in the laboratory. This will be the topic of a forthcoming publication.	[71]
Mechanical loading method	Mica	The fabricated nanopores are geometrically asymmetric, which is like an inverted quadrangular frustum pyramid. The nanopore geometry can be engineered by finely tuning the mechanical load on the AFM tip and the scanning area. It may find potential usage as functional components in nanofluidic devices.	[52]

### 2.3. Biotic Materials

#### Manual Assembly Method

**Figure 20:**
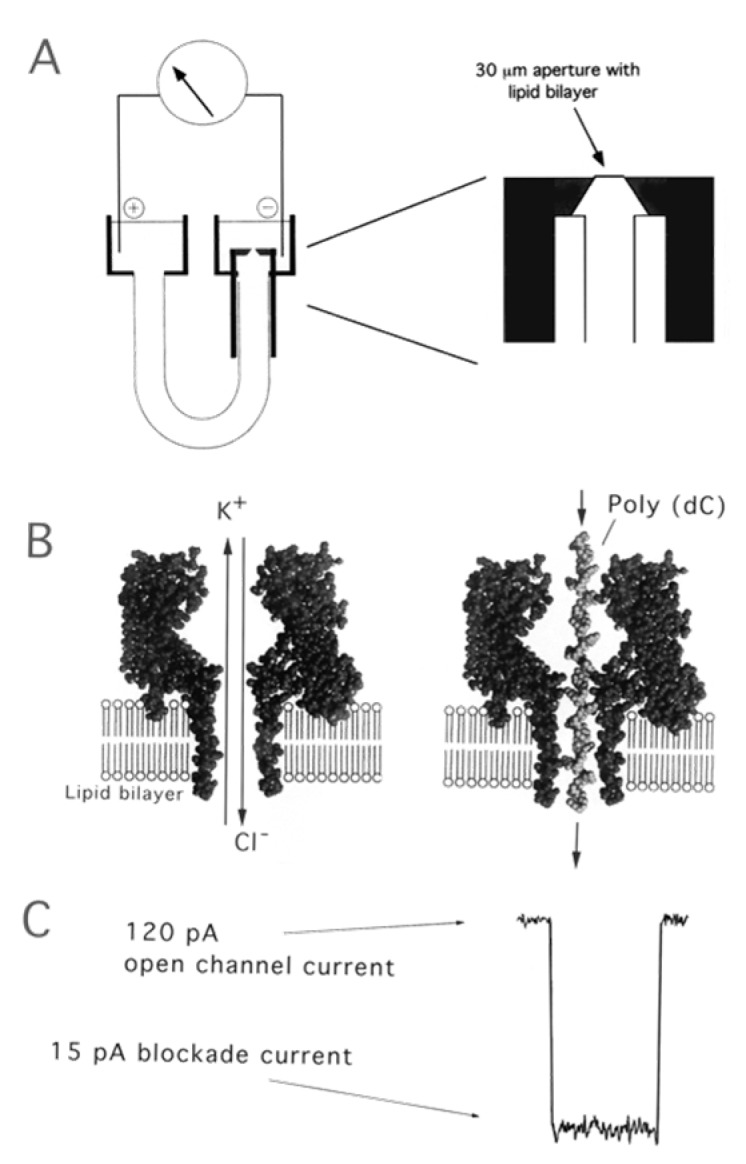
(**A**) Nanopore support device, in which a U-tube supports a lipid bilayer membrane bathed in 1.0 M KCl; (**B**) The hemolysin nanopore is shown in cross section, based on the X-ray data of Song *et al.* [146]. An ionic current of KCl is driven by the applied voltage through the open pore on the left. Under these conditions, ionic polymers such as nucleic acids are captured by the standing electrical field and driven through the pore. A synthetic poly(dC) DNA strand traversing the pore is shown on the right; (**C**) When a single-stranded nucleic acid molecule traverses the pore, a transient blockade of ionic current results, during which the ionic current is reduced from 120 to 15 pA. Reprinted with permission from reference [147]. Copyright 2002 American Chemical Society.

**α-hemolysin:** α-hemolysin is a monomeric, 33 kD, 293 residue protein that is secreted by the human pathogen *Staphylococcus aureus*. These monomers can self-assemble into a heptamer on synthetic lipid bilayers and form a 1.5 nm diameter aqueous channel across the membrane [146]. As shown in Figure 20, such single α-hemolysin channels could be introduced into lipid bilayer in the presented device with two chambers named cis and trans. The success of assembling could be monitored by measuring the transmembrane current. As soon as the current appeared, the chamber was flushed so that no further pores could insert [147]. If single-stranded DNA were to be introduced into the cis side of the bilayer, the ionic current would be blocked. Using this apparatus, we could further obtain the sequence of DNA molecules by measuring the transporting ionic current. Moreover, the bacteriophage phi29 DNA-packaging motor assembled into lipid bilayers performed similar functions as α-Hemolysin. The assembled platform showed potential application in microelectromechanical sensing, microreactors, gene delivery, drug loading, and DNA sequencing [148,149]. In the meantime, an α-Hemolysin analogue employing ultrashort single-walled carbon nanotubes (SWCNTs) was fabricated for the application in DNA sequencing [150].

**Figure 21:**
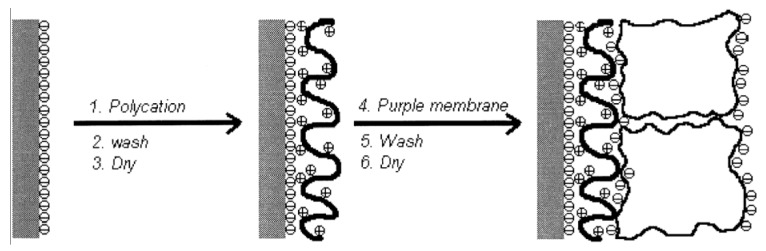
Schematic of poly (dimethyldiallylammonium chloride)/purple membrane (PDAC/PM) alternate assembly using a negatively charged solid support. Reprinted with permission from reference [151]. Copyright 1998 American Chemical Society.

**Figure 22:**
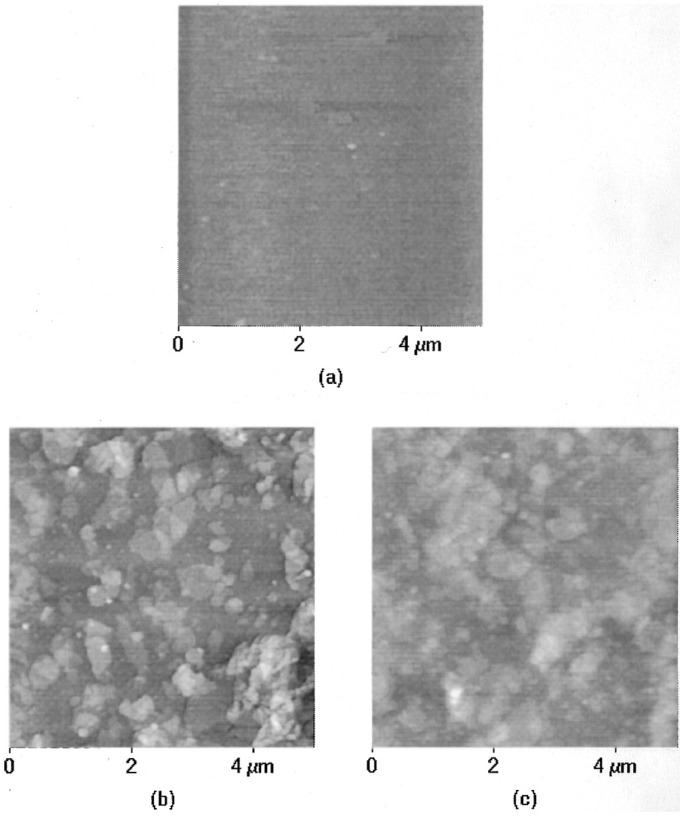
AFM images of (**a**) PDAC layer on a silicon wafer before PM adsorption; (**b**) one layer of PM adsorbed to the PDAC layer; and (**c**) two bilayers of PDAC/PM film. Reprinted with permission from reference [151]. Copyright 1998 American Chemical Society.

**Bacteriorhodopsin:** The purple membrane (PM) that contains bacteriorhodopsin is a biomaterial with great promise, and shows potential applications in many fields including optical and optoelectronic devices. For example, the cationic poly (dimethyldiallylammonium chloride) (PDAC) and PM fragments can be assembled by spontaneous alternating adsorption. Such an ultrathin composite membrane assembles schematically, as shown in Figure 21 [151]. Firstly, put a negatively charged solid support into the solution of PDAC for 5 min, and thus it can adsorb a monolayer of the polycation. Then rinse the solid support in Milli-Q water for 2 min and dry it with nitrogen. Secondly, the modified substrate is transferred into a 0.5 mg/mL PM suspension whose pH is 9.4 for 5 min, followed by rinsing with water (pH 9.4) for 2 min and drying by nitrogen again. This process is repeated until the needed number of bilayers of PDAC/PM is obtained [152,153]. The product’s AFM images of every process are shown in Figure 22.

### 2.4. Composite Materials

#### 2.4.1. Deposition Etching Method

**Figure 23:**
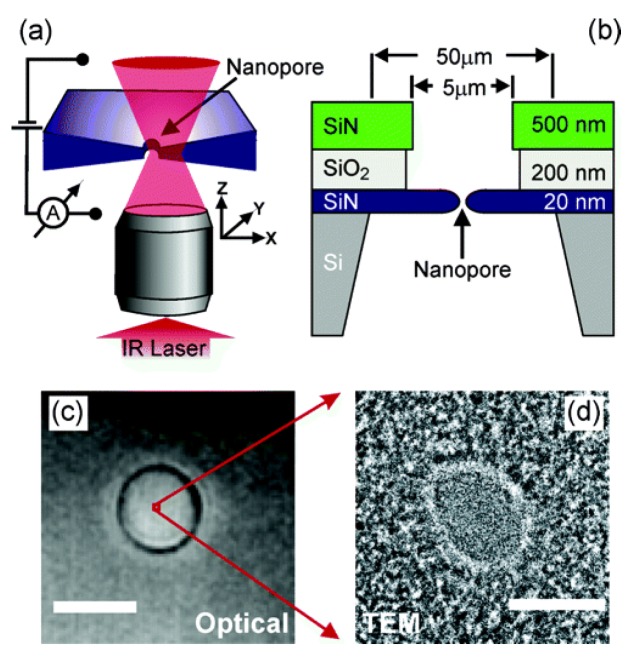
(**a**) Schematic image of the setup. The microscope objective focuses a laser into a diffraction-limited spot and the nanopore is scanned through the laser beam. The laser beam locally heats the liquid by absorption. (**b**) Section sketch of the layer structure of the completed samples with the nanopore; (**c**) optical top-view image of a SiN membrane with a diameter of 5 μm. The scale bar is 5 μm. (**d**) TEM image of a typical nanopore with a diameter of 4 nm. The scale bar is 4 nm. Reprinted with permission from reference [154]. Copyright 2005 American Chemical Society.

**SiN-SiO_2_-SiN-Si:** The schematic diagram of fabricating nanopores on composite material is exhibited in Figure 23a, which shows the essential components of the experimental configuration. The nanopores are mounted onto a home-built inverted microscope with a water immersion objective. A collimated infrared laser overfills the back aperture of the objective and the nanopore position relative to the diffraction-limited focus. Low-stress silicon nitride (SiN) membranes with thickness of 20 nm are applied to prepare nanopores whose diameters are smaller than 10 nm (Figure 23b). Before fabrication, a sandwich layer composed of a 20 nm thin SiN layer, 200 nm of SiO_2_, and 500 nm of SiN on silicon is prepared using the low-pressure chemical vapor deposition technique [154]. In order to remove the top two layers, the sandwich membrane is immersed in reactive ion etching and hydrofluoric acid at the center region with a diameter of 5 μm. The SiN membrane with thickness of 20 nm is irritated by electron beam to get a nanopore by using TEM. Optical microscopy image of the membrane in solution in Figure 23c was obtained with a CCD camera. A TEM image of a typical nanopore of 4 nm, which equals 1/1000 of the membrane diameter, is illustrated in Figure 23d. A single pixel in Figure 23c amounts to 100 times the area of the TEM image in Figure 23d [106,155].

#### 2.4.2. Reactive Ion Etching (RIE) Method

**Figure 24:**
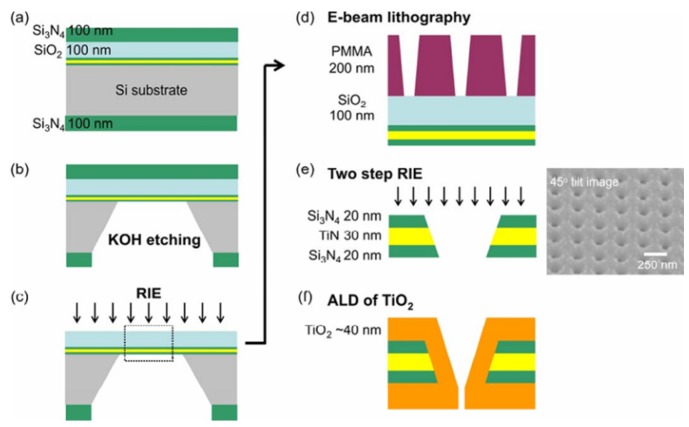
The process flow for the nanopore fabrication and the 45° tilted SEM image of the nanopore structures. Reprinted with permission from reference [156]. Copyright 2009 American Chemical Society.

**TiO_2_-TiN-Si_3_N_4_:** According to Figure 24, fabrication of sub-10 nm multiple-nanopore structures is described as follows [156]: Nanopores of molecular-level size were prepared by using E-beam lithography and atomic layer deposition (ALD) methods [157,158]. A 30 nm TiN layer was placed between two 20-nm dielectric Si_3_N_4_ films to form a sandwich structure. E-beam lithography and reactive ion etching (RIE) processes were used to form small nanopores less than 10 nm. The resolution of the top-down drilling method depends on many factors, such as beam scattering [159], resist chemistry [160], and critical dimension loss during pattern transfer [161], which is adverse for fabricating sub-10 nm nanopores. The ALD method was used to shrink the pore size by depositing controllable film on the nanometer scale [157,162]. Furthermore, the self-limiting process of the precursor molecules makes it unsuitable for preparing uniform nanochannel structures.

## 3. Conclusions and Outlook

The increase in relevant publications in this field clearly demonstrates that the design and development of 1D nanochannel materials provide an indispensable platform on which to construct a diverse biomimetic intelligent apparatus. It is an emerging field in many respects, but is still in the early stages. Inspired by the biological nanochannel in nature, researchers have selectively chosen different fabrication methods in various functional materials to obtain the artificial 1D nanochannels, such as polymers, inorganics, biotics, and complexes. In addition, our scientific community has commenced the preparation of biomimetic 1D nanochannels with various shapes. The ability to tune and control the structure of the 1D nanochannel materials offers a burgeoning platform for exploiting them in nanotechnology and materials science. However, there still exist many limitations of current fabrication methods from the biological nanochannels, such as the fabricated nanochannel showing less precision control of its states or configuration, it not being as smart as its natural counterparts in responding to external stimuli, and the fact that it does not stand as a module to be integrated into a system.

In order to pursue the controllable nanostructure of a 1D nanochannel, further properties need to be taken into account, such as the accurate nanochannel morphology characterization of various nanochannels, and analysis and interpretation of the peculiar features from different materials. Furthermre, the functionalization of the fabricated nanochannels, which has already been worked on by a number of groups, would definitely accelerate the development of this field. Therefore, utilizing the nano-size structure could lead to exceptional performance exhibited in various nanochannel materials. Additionally, it is still a prerequisite for successful implementation of ideal nanofluidics to make the nanochannel structure stable and controllable. An exciting development in the near future may be anticipated based on the design and preparation processes and experimental results summarized in this review. In future development, it will be vital to further advance the nanofabrication technology for various shapes of the 1D nanochannel and, more importantly, to amplify the efforts to build more functional 1D nanochannels with diverse membrane materials.
